# The "Two-Faced" Effects of Reactive Oxygen Species and the Lipid Peroxidation Product 4-Hydroxynonenal in the Hallmarks of Cancer

**DOI:** 10.3390/cancers2020338

**Published:** 2010-03-30

**Authors:** Stefania Pizzimenti, Cristina Toaldo, Piergiorgio Pettazzoni, Mario U. Dianzani, Giuseppina Barrera

**Affiliations:** Department of Medicine and Experimental Oncology, Section of General Pathology, University of Turin, Corso Raffaello 30, 10153 Turin, Italy; E-Mails: stefania.pizzimenti@unito.it (S.P.); cristina.toaldo@unito.it (C.T.); piergiorgio.pettazzoni@unito.it (P.P.); marioumberto.dianzani@unito.it (M.U.D.)

**Keywords:** oxidative stress, lipid peroxidation, ROS, 4-hydroxynonenal, carcinogenesis, hallmarks of cancer

## Abstract

Reacytive Oxygen Species (ROS) have long been considered to be involved in the initiation, progression and metastasis of cancer. However, accumulating evidence points to the benefical role of ROS. Moreover, ROS production, leading to apoptosis, is the mechanism by which many chemotherapeutic agents can act. Beside direct actions, ROS elicit lipid peroxidation, leading to the production of 4-hydroxynoneal (HNE). Interestingly, HNE also seems to have a dual behaviour with respect to cancer. In this review we present recent literature data which outline the "two-faced" character of oxidative stress and lipid peroxidation in carcinogenesis and in the hallmarks of cancer.

## 1. Oxidative Stress and Lipid Peroxidation, an Introduction

Oxidative stress arises from an imbalanced redox status between the production of Reactive Oxygen Species (ROS) and the biological system able to remove them. ROS, including superoxide (O_2_^−^), hydroxyl radical (·OH) and H_2_O_2_, are constantly generated in aerobic organisms. The endogenous sources usually are oxidative phosphorylation, P450 metabolism, peroxisomes and inflammatory cell activation [1,2]. Classically, ROS were regarded as host defending molecules released by the neutrophils for destroying exogenous pathogens such as bacteria. ROS can be also produced as a consequence of ionizing radiation (IR), chemotherapeutic drugs and environmental exposure to transition metals and chemical oxidants [1,3,4]. Cellular antioxidant defense enzymes, against oxidative stress, comprise the superoxide dismutases, glutathione peroxidase and catalase [1]. Superoxide dismutases and glutathione peroxidases, which are present in cytosol and mitochondria, reduce the superoxide anion to hydrogen peroxide and water, and removes the majority of hydrogen peroxide, respectively. Meanwhile, catalase, located in peroxisomes, also removes high levels of hydrogen peroxide. Nonenzymatic antioxidants, like vitamin E, vitamin C, β-carotene, glutathione, and coenzyme Q function to quench ROS [5].

ROS can randomly react with lipids, proteins and nucleic acids causing oxidative stress and damage in these macromolecules, leading to pathogenesis of age-related and chronic diseases, which include cancer, cardiovascular disease, diabetes, chronic inflammation, and neurodegenerative disorders [6,7,8,9,10,11]. Recently, a lot of evidence indicates that ROS play a central role in the key intracellular signal transduction pathway for a variety of cellular process [12].

**Scheme 1:**
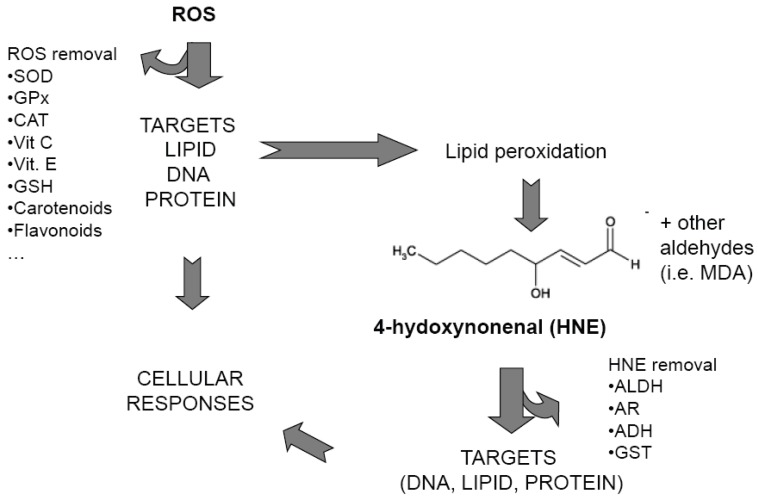
Simplified scheme showing ROS and HNE pathways. SOD: superoxide dismutase; GPx glutathione peroxidase; CAT: catalase; Vit C: Vitamin C; Vit. E: Vitamin E: GSH: Glutathione; MDA: malonildialdehyde; ALDH: aldehyde dehydrogenase; AR, aldose reductase; ADH, alcohol dehydrogenase; GST: glutathione S-transferase.

When ROS target lipids, they can initiate the lipid peroxidation process, a chain reaction that produces multiple breakdown molecules, such as malonaldehyde (MDA) and 4-hydroxyalkenals [13] (Scheme 1). 4-Hydroxynonenal (HNE), a 4-hydroxyalkenal, is the most intensively studied aldehyde [14]. HNE has three main functional groups: the aldehyde group, the C=C double bond and the hydroxyl group, which can participate, alone or in sequence, in chemical reactions with other molecules [13]. HNE is a highly electrophilic molecule that easily reacts with low molecular weight compounds, such as glutathione, with proteins and, at higher concentration, with DNA and acts as mediator of oxidative stress [13,15]. Once formed, HNE is rapidly degraded by three major reactions: reduction to 1,4-dihydroxy-2-nonene by alcohol dehydrogenases, oxidation to 4-hydroxy-2-nonenoic acid by aldehyde dehydrogenase, or formation of the glutathione-conjugate (GS-HNE) catalyzed by glutathione-S-transferases. The majority of HNE is metabolized through forming GS-HNE [16]. The half-life of HNE has been studied in several cell types, in subcellular organelles, and even in whole organisms [reviewed in 17]. Liver tissue generally has the highest capacity to metabolize HNE. Rat hepatocytes (10^6^ cells/mL) were able to metabolize about 90% of 100 µM HNE within 3 min [18]. In other cells, the HNE metabolism is not so fast, but still very efficient. For instance when 10 µM HNE was added to 10^6^ K562 or HL-60 leukemic cells, the aldehyde disappeared within 1 hour [19]. On the basis of such experimental evidence, one can conclude that HNE, even at very high lipid peroxidation rates, cannot accumulate in an unlimited manner.

HNE has been detected *in vivo* in several pathological conditions, which entail increased lipid peroxidation, including inflammation, atherosclerosis, chronic degenerative diseases of the nervous system, and chronic liver diseases, reaching a concentration up to about 10 µM [20]. However, under physiological conditions, HNE can be found at low concentrations in human tissues and plasma(0.07–2.8 µM) [13,17] where it participates in the control of biological processes, such as signal transduction, cell proliferation and differentiation. Indeed, HNE, similarly to ROS, plays an important role in controlling the intracellular signal transduction pathways involved in a number of cell responses [17,21,22,23].

## 2. ROS and HNE in Carcinogenesis

Most carcinogens are able to interact with DNA, forming *carcinogen*-*DNA* adducts. ROS and HNE seem to share this feature and this has been proposed as the mechanism of tumor induction. ROS cause oxidative modification of DNA bases resulting in DNA modification and gene mutation, which may be all be carcinogenic [24]. Hydroxy radicals, for example, react with guanosine to form 8-hydroxy-2’deoxyadenosine (8OHdG), which may lead to a G:C to T:A transversion-type point mutation contributing, for example to generation of p53 mutations. This is one of the best pieces of evidence indicating that oxidative stress is intimately associated with carcinogenesis [25]. In addition to nuclear DNA ROS-mediated, mutations in mitochondrial DNA have recently emerged as another important target in carcinogenesis [26].

However the ROS-induced association with cancer is controversial and it is unlikely that the pro-carcinogenic effects of ROS are all due to elevated direct oxidative damage to DNA bases, simply because there are situations where 8OHdG levels can be elevated, but cancers do not increase [27]. For example, patients with rheumatoid arthritis or with type 2 diabetes show elevated 8OHdG levels, but no clear evidence for a generalized increased cancer has been found [28]. This may suggest that oxidative DNA base damage alone is insufficient to cause cancer development or perhaps that damage over only a certain range is effective, excessive damage having an anti-cancer effect by promoting apoptosis [28].

The genotoxic and mutagenic effect of HNE has been demonstrated in both procaryotic and eukaryotic cell lines. This strongly alkylating aldehyde is able to directly affect DNA. The genotoxic property of HNE was demonstrated, for example, in primary hepatocytes and cerebral microvascular endothelial cells, where an increase of micronuclei, chromosomal aberrations and sister chromatid exchanges was observed [29].

Mutagenicity of HNE has been demonstrated in several experimental settings [30,31,32,33]. Possible mechanisms for HNE induced mutagenicity are the formation of DNA-adducts with metabolites of HNE. Indeed, HNE can be further metabolized to an epoxyde intermediate that interacts with DNA to form exocyclin etheno-modified DNA bases [34]. In addition, bulkly exocyclin propane-type DNA adducts with guanine, and 6-(1-hydroxyhexanyl)-8-hydroxy-1,N2-propano-2’deoxyguanine (HNE-dG) adducts may be formed [35].

Etheno-DNA adduct levels were found to be significantly elevated in organs with diseases related to a persistent inflammatory process that can lead to malignancies, such as chronic pancreatitis, ulcerative colitis, viral Hepatitis B and Crohn’s disease [36]. These authors suggested that the etheno-DNA adducts may serve as potential markers for assessing progression of inflammatory cancer-prone diseases [36].

Formation of DNA lesions by excess ROS and lipid peroxidation end products leads to an increased p53 mutation load in non-tumorous liver tissues of Wilson Disease and hemochromatosis patients [37]. This study also reported a hot-spot mutation, a G:C versus T:A transversion in codon 249 (-AGG*-). Most interestingly, it was shown that in the wild-type p53 cell line, HNE caused a high frequency of the same G vs T mutation at codon 249. So, HNE may be involved in etiopathogenesis of human cancers having a p53 mutation at codon 249, which is a unique mutational hot-spot in hepatocellular carcinomas [38]. In addition to epsilon-adducts, exocyclic 1,N2-propano-2’deoxyguanine adducts carrying a hydroxy-hexanyl side chain (HNE-dG) have appeared in HNE-treated cells [39] which seem to be involved in inducing mutation on the p53 gene [40].

The demonstration of HNE mutagenicity “*in vitro*” obviously doesn’t assure that this mechanism also occurs “*in vivo*”. Moreover, it is necessary to pay attention to HNE doses employed for such studies. Several mutagenic assays with HNE have been performed with high doses of HNE (more than 100 µM). It seems rather unlikely that HNE or other aldehydes can reach overall concentrations in the range of 100 µM in cells and organs [13]. It is conceivable that such levels may be built up locally near or within peroxidizing membranes for a short time because of their high lipophilicity. It has been calculated, for example, that the concentration of HNE in the lipid bilayer of isolated peroxidizing microsomes is about 4.5 mM [41]. Nevertheless, a convincing demonstration that this very high concentration can reach into the cells has remained elusive. On the other hand, when HNE diffuses out from membranes, its concentration is reduced by the surrounding aqueous phase. Moreover, the cytosolic HNE-metabolizing enzymes destroy HNE produced in excess so that the steady-state HNE concentration into the cells is reached quickly, ranging from 0.07 µM to 2.8 µM [13,17].

## 3. Role of ROS and HNE in the Hallmarks of Cancer

### 3.1. Regulation of Cell Proliferation

The role of ROS in cell proliferation control has been long studied by several research groups. Usually, ROS can elicit a broad spectrum of responses depending on the magnitude of the level and the duration of exposure. In general, low levels of ROS are mitogenic and promote cell proliferation, while intermediate levels cause transient or permanent cell cycle arrest and induce cell differentiation. High levels of ROS are detrimental and induce cell apoptosis or necrosis [42,43]. These actions have been ruled out by ROS action, acting as a primary messengers in the intracellular signalling cascades, such as the mitogen-activated protein kinases (MAPKs), phosphatidylinositol-3-kinase (PI3K)/Akt pathway, phospholipase C-g1 (PLCg1) signalling, protein kinase C, p53 signaling, ataxia-telangiectasia-mutated (ATM) kinase, nuclear factor-kappaB (NF-κB) signalling, and Jak/Stat pathway [2,43,44,45]. The cell response depends on the pathway activated by ROS, and that can tend to enhance survival or promote cell death. Hence, the battle for survival from cell death is determined by the relative balance among the activities regulated, the molecular background of cells and tissues, the location of ROS production, the concentration of individual ROS species and the antioxidant concentration in the cells [46]. Through distinct signal transduction cascades, ROS can induce the expression of families of heat shock proteins, immediate early genes of the bZip family members like c-Jun and c-Fos, hypoxia inducible factor, and antioxidative enzymes which help to regulate redox homeostasis, the expression of transforming oncoproteins and growth factors [46].

As far as it regards the effect of HNE in controlling cell proliferation, the majority of research reports indicate that HNE elicits a reduction of cell proliferation. The first experiments were done by using cultivated human leukemic cells, which do not contain endogenous HNE, as lipid peroxidation in such cells is practically null. When added to such cells, however, HNE disappears within 15–20 min, partially due to its binding to cell structures, partially to metabolism. So, it is sometimes necessary to continuously repeat the treatment in order to stabilize the effects [47]. With K562 cells, originally derived from a human erythroleukemia, HNE was found not only to strongly decrease cell proliferation, but also to block the expression of the oncogene c-myc which is highly expressed in untreated cells [48]. Similarly, in human HL-60 leukemia cells, 1 µM HNE strongly decreases cell proliferation, and, at the same time, blocks the expression of the oncogenes c-myc [47,49]. The inhibition of c-myc expression by HNE has also been observed in U937 and ML1 human leukemic cells, and in MEL murine erythroleukemic cells [50,51]. 

These actions of HNE were transient, but could be stabilized by a continuous supply of 1 µM HNE, repeated 10–12 times. Deeper investigations in HL-60 cells showed that the proliferation blockage occurred at the level of the G0/G1 stage of the cell cycle. In fact, after HNE treatment, there was a big accumulation of cells in this phase and a decrease of S-phase cells. This means that the progression to the S phase of the cycle is prevented [52]. Further experiments showed that most of the HNE activity on the cell cycle is affected through the inhibition of the cyclin expression, especially of cyclins D2, D1, and A [53]. As a consequence, pRB was found hypophosphorylated and the E2F transcription factor remained bound to the pRB protein [54]. Moreover, HNE reduced the expression of E2F4 transcription factor [54]. 

In tumor cells which express wild type p53, such as ML-1 human leukemic cells [50] and SK-N-BE human neuroblastoma cells [55], HNE is able to induce p53 expression which, in turn, can regulate cell cycle arrest or apoptosis induction. 

In solid tumor cells, such as 7777 and J42 hepatomas, inhibitory effects of HNE on cell proliferation are lower, probably due to the presence of a more efficient system removing the aldehydes. Indeed, these cells display a very high expression of aldehyde dehydrogenase 3, that is able to destroy a large amount of the added aldehyde [56,57]. Its inhibition by antisense oligonucleotide has strong inhibitory effects on cell proliferation, suggesting that this aldehyde plays an important role in this inhibition [58].

In other tumor cell lines, such as human neuroblastoma cells SK-N-BE cells, HNE was also able to block proliferation and induce apoptosis [55]. Moreover, HNE increased the expression of the p53 family members (p53, p63, p73) as well as the expression of the cyclin/CDK inhibitor p21 and the proapoptotic Bax. An increase in p53 expression also has been found in germ cells where HNE treatment inhibited proliferation [59]. Different research groups demonstrated that HNE inhibited proliferation of human colon tumor cells through regulation of the MAPs kinase pathway [60,61] or through the PPAR gamma pathway [62]. Moreover, a strong inhibition of cell proliferation was reported also in breast cancer cells (MCF7) treated with conjugated linoleic acid (CLA), which increases the endogenous levels of HNE [63] and in human osteosarcoma cells treated with HNE [64]. Interestingly, the effect of HNE on normal cells proliferation is more variable if not opposite to that observed in tumor cells. Concerning the atherogenic role of oxidized low-density lipoprotein and the lipid oxidatation products, it has been reported that HNE induced vascular smooth muscle cell proliferation [65,66]. More recently, other authors have shown that the proliferation rate of smooth muscle cells (SMCs) depends on HNE incubation time and concentration: a prolonged treatment with 0.1 µM HNE resulted in an increase of cell growth in young SMC but displayed cytotoxicity in aged SMCs [67]. In the same cell model Vindis and collaborators [68] demonstrated that short-term incubation of SMCs with oxLDLs and HNE induced platelet-derived growth factor receptor (PDGFR) β activation, while long-term incubation triggered a desensitization of PDGFR to its own agonist, with a progressive inhibition of PDGFR β phosphorylation. These authors concluded that PDGFR-β is a target for HNE and its progressive inhibition may contribute to defective SMC proliferation. 

A direct comparison between the HNE effect on the growth of human lymphatic leukemia cells and normal human peripheral blood lymphocytes has been done by Semlitsch and collaborators [69], which demonstrated that HNE showed a cytotoxic effect and reduced DNA synthesis in lymphatic leukemia cells, whereas it did not shown any significant toxicity on normal lymphocytes. 

These data indicate that HNE strongly reduces the proliferation of tumor cells but it increases or does not affect proliferation of normal cells in relation to the dose of HNE and the time of exposure. This dual effect may be due to the presence of aldehyde metabolizing enzymes or the high antioxidant concentration in normal cells. 

*3.2. .Regulation of Apoptosis*

It is well known that ROS induce cell death, including apoptosis. Many antitumor agents, such as vinblastine, cisplatin, mitomycin C, doxorubicin, camptothecin, inostamycin, neocarzinostatin and many others exhibit antitumor activity *via* ROS-dependent activation of apoptotic cell death, suggesting potential use of ROS as an antitumor agent [70]. Thus, a unique anticancer strategy named "oxidation therapy" has been developed by inducing cytotoxic oxystress for cancer treatment. Both death receptor- and mitochondria-mediated apoptosis depend a lot on ROS [71]. Fas ligand (FasL) triggers a rapid formation of ROS that mainly derive from NADPH oxidase as an upstream event of apoptosis induction. Moreover, FasL-induced ROS mediate the ubiquitination and subsequent degradation by proteasome of FLICE inhibitory protein (FLIP) to further enhance Fas activation [72].

Several researchers have shown that ROS related apoptosis is processed by the mitochondrial pathway, through the activation of p38 [73,74,75]. It has been demonstrated that ROS-dependent activation of MAPKs is crucial for mitochondria-mediated apoptosis [76]. In particular, c-Jun NH2-teminal kinase is the major signaling molecule mediating the ROS-induced opening of the PT pore complex [44].

In addition to direct activation of signalling pathways, ROS can promote apoptosis *via* generation of lipid peroxidation products, among them HNE plays an important role. For example, it has been demonstrated that polyunsaturated fatty acids (PUFAs) such as arachidonic, linolenic and linoleic acids markedly enhance the DNA fragmentation induced by glutamate. Thus the DNA fragmentation seems to be mediated by ROS or ROS-mediated lipid peroxidation [76]. Moreover, it has also been suggested that lipid peroxidation might proceed not only in plasma membranes but also in the nuclear membranes close to chromosomes, due to the loss of membrane integrity in cell membranes consisting of phospholipids and thereby may make circumstances suitable for oxygen radicals to attack chromatin and DNA [76]. However, more convincing data are provided from the studies about the effects of the lipid peroxidation product, HNE, in inducing apoptosis in several cell models. Awasthi and collaborators [77] demonstrated that the overexpression of the α class of glutathione-S-transferases leads to a lower steady-state level of HNE, and induces resistance to apoptosis initiated by lipid peroxidation causing agents such as H_2_O_2_, UVA, superoxide anion and pro-oxidant xenobiotics. The proapoptotic action of HNE has been directly demonstrated in neuronal cells [78], in vascular smooth muscle cells [67] and in lens epithelial cells [79]. Furthermore, the proapoptotic effect of HNE, accompanied by an increase in Bax protein, has been demonstrated in SK-N-BE human neuroblastoma cells [55] and in CaCo-2 human colon cancer cells [62]. 

The mechanisms of HNE-induced apoptosis have been studied also by de Villiers and collaborators [80]. These authors found that HNE caused dose-dependent apoptosis in two rat hepatic stellate cell lines, HSC-T6 and CFSC-2G cells. Apoptosis in HSC-T6 cells was associated with increased mRNA and protein expression of the pro-apoptotic adaptors/regulators FasR, FasL, Bax, and caspases-2 and -3. In contrast, CFSC-2G cells showed no changes in FasR, Bax and caspase-3 mRNA levels. Other authors studied the mechanisms of the apoptotic response to HNE by analysing MAP kinase and caspase activation pathways in 3T3 fibroblasts [81]. HNE induced early activation of JNK and p38 proteins but down-regulated the basal activity of ERK-1/2. Moreover, HNE induced the release of cytochrome c from mitochondria as well as caspase-9 and caspase-3 activation. Overexpression of dominant negative c-Jun and JNK1 in 3T3 fibroblasts prevented HNE-induced apoptosis, which indicated a role for the JNK-c-Jun/AP-1 pathway. These data confirm previous observations from Biasi and collaborators [82] who found that c-Jun N-terminal kinase upregulation was a key event in the proapoptotic interaction between transforming growth factor-β1 and 4-hydroxynonenal in colon mucosa [82]. Exposure of vascular smooth muscle cells to HNE showed augmented apoptotic changes in a concentration-dependent manner and was associated with an increased production of ROS. These authors conclude that mitochondrial dysfunction plays a key role in mediating HNE-induced vascular smooth muscle cell apoptosis through an increased mitochondrial production of ROS [67]. The involvement of HNE-mediated apoptosis in the oxidative damage of neural cells has been suggested by several authors [78,83] by analyzing the role of HNE in cell death resulting from β-amyloid exposure. These authors showed that HNE treatment of neuronal cells induced dose-dependent death and caspase activation which were blocked by inhibition of caspases. 

An indirect demonstration of the ability of HNE to induce apoptosis is represented by results obtained by lowering the intracellular concentration of HNE in cells transfected with enzymes involved in HNE metabolism. For example, the over-expression of GSTA4-4 or GST5.8, which detoxify HNE, inhibits apoptosis induced by the xantine/xanthine oxidase, H_2_O_2_, UV and doxorubicin [84]. HL-60 cells also acquire resistance to apoptosis induced by 20 µM HNE when transfected with GSTA4-4n [85]. On the contrary, increased concentration of HNE, obtained by inhibiting RalBP1-mediated transport of GS-HNE, leads to apoptosis in cells of various origin even in the absence of any stressor [86,90]. In conclusion, several reports, by showing a role for HNE in apoptosis induction, demonstrate the involvement of lipid peroxidation in this process.

### 3.3. Regulation of Replicative Potential of Cells

It is widely accepted that the replicative potential of the cell is limited. After a certain number of division cells start the senescence or permanent irreversible replication arrest [91]. This status occurs when the cell encounters sublethal levels of certain stresses, such as elevation of ROS, oncogene overexpression, or irradiation. It has been shown that treatment with exogenous reactive oxygen species, such as H_2_O_2_, induces premature senescence [92,93]. In fact, nearly all stresses that induce senescence are thought to increase intracellular ROS [94]. It was broadly believed that high oxygen levels (hyperoxia) should elevate intracellular ROS production. Indeed, hyperoxia can force human fibroblasts [95] and human articular cartilage chondrocytes [96] to arrest in a senescent-like-fashion. Over-expression of antioxidant genes such as superoxide dismutase or catalase and the maintenance of cell culture in a low oxygen environment extend lifespan [97]. Controversial data about the role played by ROS were obtained in human lung fibroblasts cultured under hypoxia [98]. In this condition ROS levels increase, rather than decrease, paralleling an increase of replicative life span. 

Senescence is accompanied by telomere loss. Telomeres correspond to the ends of eukaryotic chromosomes and are specialized structures containing unique (TTAGGG)n repeats, protecting the chromosomes from DNA degradation, end to end fusions, rearrangements, and chromosome loss [99]. The number of telomere repeats decreases (by 50–200 nucleotides/cell division) during aging of normal somatic cells [100] and it has been proposed to be a mitotic clock marking progression of a cell toward the end of its replicative lifespan. Synthesis and maintenance of telomeric repeats are mediated by a specialized enzyme, known as telomerase [101], a ribonucleoprotein complex which contains a catalytic subunit, the human telomerase reverse transcriptase (hTERT) and a small integral RNA component (hTR), utilized as a template for the synthesis of the dGT-rich strand of telomeres [101]. Inhibition of telomerase activity leads the cells to senescence [102]. Although normal somatic cells do not express telomerase, immortalized cells such as tumor cells express this enzyme [103,104]. For this reason, telomerase activity and hTERT expression have become new targets for tumor treatments. The effect of ROS on telomerase activity is controversial. In vascular smooth muscle cells, senescence is accelerate by oxidative stress-induced DNA damage accompanied by the inhibition of telomerase and marked telomere shortening [105]. On the other hand, it has been recently reported that oxidative stress enhanced the malignant potential of human hepatocellular carcinoma through the activation of telomerase [106]. In this field, the demonstration that, in hypoxic conditions, ROS activate the transcription factor hypoxia inducible factor (HIF), which induces transcription of the hTERT gene [98] further indicates that the role of ROS in telomerase control is not univocal. 

On the contrary, our research group demonstrated that HNE strongly inhibited the activity of telomerase and the expression of its catalytic subunit hTERT in three different human leukemic cell lines, HL-60, U 937 and ML-1 [50]. Telomerase activity can be regulated by the reduced glutathione (GSH), the natural scavenger of HNE. Indeed, Borras and collaborators demonstrated that intracellular GSH content paralleled telomerase activity in 3T3 fibroblast [107]. However, in other cells, such as colon carcinoma Caco-2 cells GSH depletion by HNE can only partially contribute to the down-regulation of telomerase activity [108].

The major mechanism by which this aldehyde exerts the inhibition of telomerase activity seems to be the regulation of transcriptional control of the hTERT gene, by modulating the expression of transcription factors belonging to the Myc/Mad/Max network. The hTERT promoter contains several binding sites for transcription factors, including activators (c-Myc, Sp-1) and repressors (Mad1) [109] and we studied the expression of these proteins and their binding to the hTERT promoter after HNE treatment. Myc protein forms dimers with the Max protein that bind to the 5’-CACGTG-3’ sequence (E-box) in promoters and transactivate target genes. Max also forms dimers with the Mad family proteins and all complexes bind to the same E-box, having an inhibitory function [110]. HNE was able to inhibit c-Myc and induce Mad-1 expression in all of the three cell lines. The E-box binding studies demonstrated that in HNE-treated HL-60 cells there is a decrease in Myc binding complexes and an increase in Mad-1 binding complexes. This modulation could contribute to the switch from c-Myc/Max to Mad1/Max occurring at the E-box of the hTERT promoter. This inhibitory mechanism of hTERT expression and, as a consequence, of telomerase activity by HNE has been confirmed in CaCo-2 human colon cancer cells also [108].

### 3.4. Regulation of Angiogenesis

In addition to the genetic and epigenetic changes that occur during transformation, another discrete step is required to allow tumor propagation and progression: the induction of a tumor vasculature, termed the “angiogenic switch” [111]. Angiogenesis is orchestrated by a variety of activators and inhibitors and, during the “angiogenic switch”, the predominant angiogenic factors can activate quiescent endothelial cells, thus stimulating them to initiate migration, proliferation and organisation in tubular structure. Activators of endothelial-cell proliferation and migration are mainly receptor tyrosine kinase ligands, such as vascular endothelial growth factor (VEGF), fibroblast growth factor (FGF), platelet-derived growth factor (PDGF). A prototypical angiogenesis inhibitor is thrombospondin-1 and many other molecules have been identified, such as angiostatin and endostatin [111]. The importance of the angiogenic program in controlling tumor growth is underlined by antiangiogenic gene therapy as a new therapeutic approach to the treatment of cancer patients [112].

A large body of evidence demonstrate the pro-angiogenic role of ROS and results have been reviewed by several authors [12,46,113,114,115]. Exogenous ROS stimulate induction of VEGF, promote cell proliferation and migration [116,117,118,119], cytoskeletal reorganization [120] and tubular morphogenesis [118,121] in ECs. Moreover, ROS are involved in the production of pro-angiogenetic factors in different cell types, both of normal and tumoral origin [46,113,122,123]. As previously reported, the major source of ROS is aerobic respiration in the mitochondria. In tumor cells and in endothelial cells, ROS can also be generated by the NADPH-oxidase [113,124]. Modulation of the activity of this enzyme affects cell growth and angiogenesis in various experimental models [125,126,127]. For example, the transfection of NIH 3T3 cells with the Nox1 gene, a homolog of gp91*phox*, the catalytic subunit of the NADPH-oxidase, renders them capable of forming well vascularized tumors, whereas the parent cells form microscopic dormant tumors that are poorly vascularized. Similarly, Nox1 expression converts DU-145 prostate epithelial cells from weak to strong tumorigenic potential, with a corresponding increase in tumor vascularity, pointing out the generality of the angiogenic effect of Nox1 [128].

The angiogenic property of HNE is under investigation, but literature is still poor and conflicting results have been reported. HNE, at concentrations of 1.0 and 2.5 µM, significantly stimulated rat aortic smooth muscle cell growth and PDFG production [129]. Ayalasomayajula and Kompella reported an increase of VEGF expression in human retinal pigment epithelial cells after treatment with 1 µM HNE [130]. Moreover VEGF receptor 1 expression was induced in a dose-response manner in myeloid cells treated with 15 and 60 µM HNE [122]. However, the anti-angiogenic properties were recently demonstrated by Stagos and collaborators in human bone marrow endothelial cells (HBMEC) [131]. These authors demonstrated that 5 and 10 µM HNE were able to inhibit the tube formation of HBMEC cells. In an attempt to elucidate the mechanism, they demonstrated that this inhibition occurs at least in part *via* HNE-induced expression of chondromodulin-I (CHM-I), a protein with well-known anti-angiogenetic properties [131]. The anti-angiogenic property of HNE is also sustained by our recent findings on microRNA (miRNA) [132], a class of conserved non-coding small RNAs, which regulate gene expression by translation repression of coding mRNAs [132]. HNE, indeed, is able to modulate the expression of ten miRNAs in HL-60 human leukemic cells. Among them, we observed the down-regulation of miR-378 [132], which has been demonstrated to enhance cell survival, tumor growth, and angiogenesis in the human glioblastoma U-87 cell line [134], through repression of the expression of two tumor suppressors, Sufu and Fus-1. In accordance to these results, we demonstrated that HNE is also able to up-regulate Sufu expression in HL-60 cells after HNE-treatment [132].

### 3.5. Regulation of Cell Adhesion, Tumor Invasion and Metastasis

Tumor invasion and metastasis, responsible for most cancer death in humans, consist in a series of steps that include shedding of cells from a primary tumor into the circulation, survival of the cells in the circulation, arrest in a new organ, extravasation into the surrounding tissue, initiation and maintenance of growth, and vascularization of the metastatic tumor. All of these processes are regulated by multiple factors and must be successfully completed to permit the outgrowth of metastatic tumors in the new microenvironment [135].

The cellular disengagement requires the modulation of expression of cell-cell adhesion molecules at adherent junctions [136], such as E-cadherin, which, on its cytoplasmic face, organizes the actin cytoskeleton *via* its association with β-catenin, a transcription factor involved in signalling *via* the Wnt pathway [136].

Integrins perform essential roles in cell adhesion to the extracellular matrix (ECM). Evidence suggests that the interference with integrin-mediated adhesion can impair tumor metastasis [137]. A role for the Focal adhesion kinase (FAK) on cell interactions with the extracellular matrix in tumors has been recently demonstrated [138]. This kinase maintains a role in adhesion, cell spreading and migration and cell proliferation and is up-regulated in metastatic tumors [139].

The ECM-degrading enzymes, such as matrix metalloproteinases (MMPs) play also an important role in mediating tumor cell invasion. The general belief is that overexpression of a specific MMP, either by tumor cells or the surrounding stroma, is pro-tumorigenic [140]. However, there is an increasing amount of literature demonstrating that the expression of certain MMPs, either at the primary or the metastatic site, provides a beneficial and protective effect in multiple stages of cancer progression [141]. Similarly, the tissue inhibitors of metalloproteinases (TIMPs) are intriguing molecules that have both pro and antitumorigenic effects [142].

The extravasation process of circulating cancer cells is also a key step in metastasis which has features similar to the inflammatory recruitment of leukocytes. Indeed, this process is regulated by cell adhesion molecules (CAMs), such as intercellular cell adhesion molecule-1 (ICAM-1), vascular endothelial cell adhesion molecule-1 (VCAM-1), E-selectin, and P-selectin [143]. These molecules are usually up-regulated in tumor progression. 

It has been reported, in addition to regulating tumor growth, survival and angiogenesis, that ROS also control the formation of tumor metastases [106]. In many types of tumors including prostate cancer, melanoma and breast cancer, the increased metastatic ability of tumor cells is positively related to their intracellular ROS level [106]. Exogenously administration of ROS would enhance certain stages of metastasis [144], while anti-oxidant treatment could attenuate metastatic progress [145].

Recently, Ishikawa and collaborators [146] have provided direct evidence to confirm the causative relationship between ROS and tumor metastasis. After being replaced with mitochondrial DNA (mtDNA) derived from a higly metastatic mouse tumor cell line, an originally poorly metastatic cell line acquires the metastatic potential. The transferred mtDNA contains mutations producing a deficiency in respiratory complex I activity and are associated with overproduction of ROS. Pretreatment of the highly metastatic tumor cells with ROS scavengers suppressed their metastatic potential in mice [146]. ROS also participate in the loss of cell-cell-interaction, inhibiting the E-cadherin expression or disruption of the cadherin-β-catenin complex [147,148,149].

Besides cell-cell-interaction, the adhesion of cells in the extracellular matrix as mediated by integrins is of importance for the stability of tumor tissue [150]. ROS can have a different effect on integrins, due to their dual role. For example, it has been found that ROS lead to a significant reduction of the αvß3-integrin [151] in sarcomas, while ROS lead to up-regulation of a set of integrin family members (integrin α2, α6, and β3) in mammary epithelial cells [147]. Oxidative stress is also able to activate FAK by inducing its autphosphorylation in tyrosine and this could explain the role of ROS in mediating cell spreading and migration [152]. 

Oxidative stress may induce tumor cell migration and invasion through the activation of MMPs, also. ROS can activate MMPs, such as MMP-2, probably though the reaction of ROS with thiol groups in the protease catalytic domain [153]. Additionally, ROS are able to up-regulate the expression of several MMPs (MMP-1, MMP-3, MMP-9, MMP-10, MMP-13) [154]. The pathway proposed in this up-regulation seems to involve Ras or the MAPK family member ERK1/2, p38 and JNK [155]. The direct activation of the Ras effector ERK1/2 leads to the induction of the Ets transcription factor and JNK-regulated the transcription factors Jun and Fos which form the AP-1 complex. Both AP-1 and Ets regulate MMP expression [154,156]. Additionally, other redox-sensitive transcription factors, such as NF-κB can regulate MMPs expression [154].

According to these findings, MMP inhibitors and TIMPS, are down-regulated by ROS [157], leading to an increase of the metastatic potential of tumors. In some cases, ROS do not induce TIMP expression, while MMPs activity is enhanced, but in any case the balance between MMPs and TIMPs is shifted towards MMPs, with a metastatic significance [158,159]

However, results in this field are controversial. Indeed, the MMP-2 and 9 activities in the DS-sarcoma cell line were markedly reduced immediately after ROS-inducing treatment [151]. Moreover the Rac-dependent induction of ROS is able to inhibit TIMP-1 expression in renal carcinoma cells and other different tumor cell lines [160], inhibiting matrigel invasion [161].

ROS have been involved in the extravasation process. For example, oxidative stress regulates the expression of cell surface protein ICAM-1 in endothelial and epithelial cells, most likely due to the activation of NF-κB [162].

The effects of HNE on cell adhesion, invasion and metastasis are fragmentary and ambiguous. An anti-metastatic and anti-invasive role of HNE has been demonstrated by our research group, since this aldehyde inhibits Notch expression [163], a transmembrane receptor involved in neoplastic epithelial-mesenchymal transition [164], which promotes E-cadherin down-regulation [165].

Usatyuk and collaborators [166] demonstrated that HNE increases the vascular endothelial permeability, by affecting focal adhesion, adherence, and tight junction proteins. Indeed, exposure of bovine lung microvascular endothelial cells (BLMVECs) to HNE induced reactive oxygen species generation and altered cell-cell adhesion as measured by transendothelial electrical resistance. In particular HNE supresses tyrosine phosphorylation of FAK, and caused the redistribution of catenin, paxillin and VE-cadherin [166]. Moreover, HNE caused a decrease in surface integrin levels, without altering total α5 and β3 integrins. The formation of HNE-derived Michael adducts with specific proteins seems to be the mechanism by which this aldehyde is able to affect cell-cell adhesion since HNE-adducts were observed in FAK, E-cadherin, β-catenin and α5 and β3 integrins[166]. Similar results were obtained in human osteosarcoma cells, where HNE was able to down-regulate FAK expression [167]. Moreover, these authors reported an increase of α_5_β_1_ integrin expression after HNE-treatment [167], which may be involved in the reduction of proliferation and metastatic ability [168,169]. 

The effect of HNE on MMPs and TIMPs expression are differentially modulated by HNE, depending on cell type. In normal cells, such as vascular smooth muscle cells, MMP-2 is enhanced by HNE *via* mitochondrial ROS-mediated activation of the Akt/NF-κB signaling pathways [170]. Consistently, HNE also elicited the production of MMP-1 in human coronary smooth muscle cells, with the involvement of PDGFR-β and ERK1/2 [171]. In osteoarthritic (OA) synovial cells, HNE induces MMP-13 synthesis and activity but in contrast inhibits type II collagen and TIMP-1 synthesis playing a role in OA cartilage degradation [172].

In contrast with these findings, it has been shown that HNE did not significantly affect expression of MMP-1 and MMP-2 while it up-regulated of TIMP-1 expression in human hepatic stellate cells, as pro-fibrogenic stimulus [173].

HNE effects on cell adhesion molecules of endothelium are generally convergent, since several authors demonstrated the inhibitory role of this aldehyde on the endothelial response. For example, HNE caused a dose-dependent decrease in production of both interleukin-8 (IL-8) and intercellular adhesion molecule-1 (ICAM-1) in HUVEC [174] and, in human aortic endothelial cells, HNE dramatically inhibited the expression of several adhesion molecules, such as ICAM-1, VCAM-1 and E-selectin, induced by inflammatory stimuli, accompanied by a significant reduction of NF-κB activation [175]. 

Recently we demonstrated that HNE reduced adhesion of HL-60 cells to endothelial cells (HUVECs) [176]. The mechanism proposed involves the formation of HNE-α-enolase adduct(s) in HL-60 human leukemic cells, impairing the plasminogen-binding activity of α-enolase. 

### 3.6. Regulation of Genomic Instability-DNA Repair

An unstable genome is a hallmark feature of nearly all tumors; this instability first appears early in tumor progression, and can take several forms [177]. Intrachromosomal genomic instability in cancer arises from several mechanisms: increased rates of damage, deficiencies in the DNA repair enzymes and checkpoint defects which fail to halt the cell cycle until repair. Several of the hallmarks of cancer described above are associated with genomic instability. For instance, the ability to evade apoptosis through alterations in p53 [178,179] and Bcl-2 [180], as well as shortened telomere length [181], are all associated with increased genomic instability in breast cancers. This instability underlies the vast majority of genomic events. Chromosomal instability at the whole chromosome level arises from inappropriate segregation, recombination and the like, and generates relatively few events [177].

Inefficient DNA repair is a well documented cause for the generation of genomic instability in cancer [182]. For instance, genetic disorders such as Xeroderma pigementosum provide clear examples of how defects in these genes can contribute to genomic instability and malignancy [182,183]. Damage repair deals with single events through base excision repair (BER), nucleotide excision repair (NER), or mismatch repair (MMR). Specialized genes exist for various forms of repair optimized for the particular type of damage [184].

The major mechanism that cells use to repair oxidative damage lesions, seems to be the BER [185], while the HNE-DNA adducts seems to be repaired preferentially by NER [30].

The role of ROS and HNE in contributing to genetic instability has not yet been clarified and a double effect can arise from experimental evidences. As previously reported, both ROS and HNE induced DNA mutations through the breakdown of DNA, oxidation of DNA bases or adducts formation. ROS induced mutations in DNA can be prevented by a DNA MMR system that maintains sequence fidelity during DNA replication. Key members of the MMR system include hMSH2 and hMSH6 [186]. Both ROS and HNE can interfere with genomic instability and DNA repair. It has been demonstrated that oxidative stress interferes with repair of single base mutations, by inhibiting hMSH6 [187] and HNE could contribute to generate DNA instability by inhibiting, in human cells, the nucleotide excision repair, the most important process able to repair DNA-adducts [30]. On the other hand, ROS and HNE induce the expression of p53, the *guardian* of the genome which is able to trans activate genes whose products arrest cell cycle (p21), induce DNA repair (*gadd* 45) or promote apoptosis (Bax) [188]. 

## 4. Concluding Remarks

In this review we summarised the different effects displayed by ROS and HNE in controlling carcinogenesis, cell proliferation and tumor progression and invasion. In all these areas the effects of ROS and HNE are different, in relation to doses, cell type and molecular pathway employed. 

Moreover, for both ROS and HNE, the majority of experiments were performed “*in vitro*” and the lack of “*in vivo*” evidence limits the possibility for a real evaluation of their effects on tumor development. Indeed, in tumor tissue the presence of ROS, as well as of lipid peroxidation products, depends not only on their concentration in each tumor cell, that by virtue of the tumor heterogeneity, can be highly variable, but also on their concentration in surrounding tissue that may affect the tumor cell response. Finally, it must be underlined that both ROS and HNE are physiologically present in the cells and they play an important role in cell life. For this reason, it is very important to clarify the risks and benefits of a perturbation of ROS and HNE levels in tumor cells and in this field there is much work to be done.
